# Effects of Growing-Finishing Pig Stocking Rates on Bermudagrass Ground Cover and Soil Properties

**DOI:** 10.3390/ani10091666

**Published:** 2020-09-16

**Authors:** Silvana Pietrosemoli, Charles Raczkowski, James T. Green, Maria Jesús Villamide

**Affiliations:** 1 Department of Animal Science, College of Agriculture and Life Sciences, North Carolina State University, Raleigh, NC 27695-7621, USA; 2 Departamento de Producción Agraria, E.T.S.I. Agronómica, Alimentaria y de Biosistemas, Universidad Politécnica de Madrid, 28040 Madrid, Spain; mariajesus.villamide@upm.es; 3 Department of Natural Resources and Environmental Design, College of Agriculture and Environmental Sciences, North Carolina Agricultural and Technical State University, Greensboro, NC 27411, USA; charlesraczkowski@gmail.com; 4 Department of Crop and Soil Science, College of Agriculture and Life Sciences, North Carolina State University, Raleigh, NC 27695-7621, USA; jim_green@ncsu.edu

**Keywords:** pasture pigs, bermudagrass, stocking rates, soil sampling position, soil depth, ground cover, soil properties, soil nutrients, nutrients distribution, outdoor pigs

## Abstract

**Simple Summary:**

A common challenge for most livestock industries is to identify more productive, efficient and sustainable pasture-based production systems that have a positive effect on animal welfare, biodiversity and long-term operation profitability without negatively influencing the environment. Implementing best management practices allows producers to achieve profitability and environmental goals. Maintaining an appropriate ground cover, minimizing the use of external inputs as fertilizers and pesticides and adopting agroecological approaches are key for sustainable pasture management. Pasture-based pig production systems are considered animal welfare and environmentally friendly. However, the number of animals grazing can influence the vegetation ground cover and the amount of nutrients imported to the systems. This study compared the effects of four different pig stocking rates (37, 74, 111 or 148 pigs ha^−1^) over two 14-week grazing periods, on the vegetation ground cover and soil properties of bermudagrass paddocks. Increasing the number of animals aggravated the damage to the vegetative ground cover and raised the amount of nutrients deposited on the soil. For conservation purposes, the number of pigs grazing bermudagrass should be equal to or less than 37 pigs ha^−1^.

**Abstract:**

This study compares four stocking rates (37, 74, 111 and 148 pigs ha^−1^) for growing to finishing pigs (18.4 ± 0.5 kg and 118.5 ± 2.0 kg and 35.7 ± 2.1 kg and 125.7 ± 2.3 kg initial and final BW for grazing periods 1 and 2, respectively) and their effect on ground cover and soil traits in bermudagrass (*Cynodon dactylon* [L.] Pers) pastures, over two 14-week grazing periods (July–September and May–August). The study was conducted at the Center for Environmental Farming systems at the Cherry Research Station, Goldsboro North Carolina. A continuous stocking method was implemented to manage the pasture. The percent ground cover was estimated with a modified step point technique. Soil samples were collected in three sampling positions (center, inner and outer areas of the paddocks) and two soil sampling depths (0–30 and 30–90 cm). The experimental design was a completely randomized block with three field replicates. Data were analyzed using the PROC GLIMMIX procedure of SAS/STAT ^®^ Version 9.4. Greater ground cover and lesser soil nutrient concentrations were registered in bermudagrass paddocks managed with 37 pigs ha^−1^. The results of this study also validated the existence of a spatial pattern of soil properties, which differed among sampling positions and depths.

## 1. Introduction

A common challenge for most livestock industries is to identify more productive, efficient and sustainable pasture-based production systems that have a positive impact on animal welfare, biodiversity and long-term operation profitability without negatively affecting soil health. The way that the available resources (land, forage species, animals, infrastructure and climate) are organized and used to achieve the goals of the operation is key for sustainable animal production. Grazing management allows for more efficient use of resources by structuring how we make a better utilization of them. Franzluebbers et al. [1] listed the traits that describe well managed pasture systems. They include: maintaining an appropriate ground cover, minimizing the use of external inputs as fertilizers and pesticides and adopting agroecological approaches. Production systems having these characteristics could provide a profit to farmers while maintaining diverse ecosystem services to the community. Recently, a renewed interest in pasture-based production systems has been observed [2] but information related to sound management practices oriented toward conservation of the resources is scarce. Pietrosemoli and Green [3] indicated that pasture pig production systems are confronted with a double challenge—to produce quantity and quality of forage year-around and to assure forage persistence. Under this approach, the list from Franzluebbers et al. [1], could be complemented with aspects pertinent to pasture pig systems to incorporate: minimizing the soil nutrient load, enhancing the dispersal of soil nutrients along the paddocks and reducing soil compaction.

Management strategies have a direct effect on the environmental outcome of pasture-based livestock operations. Stocking rates particularly may influence animal grazing behavior and performance as well as pasture characteristics and soil properties [4,5,6]. Profitability has been associated with the number of animals per unit area or stocking rate. Therefore, intensification of the grazing management via the implementation of high stocking rates has been considered a way to increase production and profitability of livestock operations but this strategy could involve environmental risks, especially in pasture pig systems. Pasture pigs have a better opportunity to express natural behavior compared to pigs raised indoors [7]. Nonetheless, if left uncontrolled behavior expressions such as grazing, rooting, trampling, wallowing and selecting dunging areas could trigger damages to the vegetation and the soil. Reduced ground cover leaves the soil exposed to weather elements and prone to erosion. Additionally, high soil nutrient concentrations in selected dunging areas could surpass the utilization capacity of the vegetation, increasing nutrient losses and becoming a water pollution source [3]. In contrast, areas with lesser nutrient loads may lead to reduced fertility.

The objectives of this study were to evaluate the effect of growing to finishing pig stocking rates on the vegetative ground cover, the soil properties and to establish the existence of a spatial pattern of soil properties in bermudagrass (*Cynodon dactylon* [L.] Pers) paddocks grazed with pigs during two 14-week periods, thus providing for a better understanding of soil nutrient dynamics, leading ultimately to the improvement of the management practices.

## 2. Materials and Methods 

### 2.1. Experimental Site Description

The grazing studies were conducted at the Center for Environmental Farming System (CEFS, Cherry Research Station, lat. 35°24′5″ N, long. 78°1′53″ W) in Goldsboro North Carolina, USA. Three soil taxonomies were identified: Johns sandy loam (fine-loamy over sandy or sandy-skeletal, siliceous, semi-active, thermic Aquic Hapludults), Kenansville loamy sand (siliceous, sub-active, thermic Arenic Hapludults) and Lakeland sand loamy (Thermic, coated Typic Quartzipsamments) [8]. The landscape was almost flat with less than 1% slope. The climate of the area is classified as humid subtropical (Trewartha climate classification) with warm and humid summers and mild winters and occasional snow. In this climate, the average total annual precipitation is 1465 mm, the average temperature varies between 10.8 and 22.5 °C and the growing season generally extends from early May to mid-October. The experiment was replicated twice, two pig grazing periods, 1st: July to September (Year 1) and 2nd: May to August (Year 2), while maintaining the same paddock conformation and field replicates. The paddocks were allowed to rest for 32 weeks between pig groups. 

### 2.2. Forage

One hectare of well-established (10+ year) bermudagrass previously intensively grazed with dairy cattle was used for the experiment. Three blocks were demarcated using a temporary electric fence. In each block, four square-shaped paddocks were proportionally sized to equal the stocking rates under evaluation. The four stocking rates were 37, 74, 111 and 148 pigs ha^−1^ and the paddocks size ranged between 1350 m^2^ and 340 m^2^ with a grazing pressure between 10,756 and 2689 kg of body weight ha^−1^ (Table 1). The paddocks contained a shelter, a feeder and a drinking and cooling station. The shelter, without flooring and the drinking/cooling station were located in the center of the paddock, while the feeders were positioned along one of the fences to facilitate feed delivery. Service structures were kept in the same emplacement during both pig grazing periods. Groups of pigs were randomly assigned to each paddock, which was managed under a continuous stocking system. Paddocks were winter fallow.

#### Vegetative Ground Cover Estimation

Ground cover was estimated weekly following a modified step-point method [9] along transects lines evenly spaced in each paddock. The presence of living vegetation, vegetation residues and bare soil were recorded every three steps on 6, 7, 9 and 12 transects 18.35, 21.21, 26.0 and 36.58 m long, for the paddocks managed with 148, 111, 74 and 37 h ha^−1^, respectively. The transects were identified using 1.20 m PVC pipes (1.3-cm diameter) permanently located in two parallel lines at the sides of the paddocks. Ground cover was estimated adding living vegetation plus vegetation residues cover. Ground cover data were recorded in weeks 1, 5, 10 and 14 during both grazing periods.

### 2.3. Animals

All animal related methods and protocols followed the guidelines developed by the North Carolina State University Institutional Animal Care and Use Committee (IACUC 09-021-A). A total of 120 pigs (castrated male and female) were included in the experiment. Animals used in the first grazing period (July to September, 14-week) were different from those used in the second period (May to August, 14-week). The first batch of pigs included 60 purebred Yorkshire (18.4 ± 0.5 kg and 118.5 ± 2.0 kg initial and final BW, respectively) that had been born and raised at the CEFS swine unit in a deep straw system. The second group (35.7 ± 2.1 kg and 125.7 ± 2.3 kg initial and final BW, respectively) was genetically more heterogeneous, with pure Yorkshire from CEFS (37% of the animals used) and crosses (Landrace × Duroc) and (Landrace × Hampshire) bought at a commercial operation. Pigs were not nose-ringed. Before starting the experiment, both groups of pigs were kept in an outdoor training paddock for two weeks, to let them get accustomed to the new environment and electric fences. Pigs were allotted to 12 groups of five pigs each such that all the groups had the same average total initial weight. The groups were then randomly assigned to the paddocks. Pigs on all groups were managed in a similar manner. 

In the paddocks, pigs had access to an open shelter structure, a nipple drinker and a two-opening self-feeder. The creation of wallow areas was not encouraged but a water valve was installed in the top of the pipe where the drinker was mounted and was used as a cooling station during the hottest times of the day. Pigs had *ad libitum* access to a home mix grain mixture (containing corn, soybean, minerals and vitamin mixes averaging 168 g kg^−1^ crude protein and 2463 kcal kg^−1^ net energy) and were individually weighed at the beginning and at the end of the grazing period, in the morning, without feed or water withdrawal. Feed offered was weighed twice a week for each paddock and feed leftover in the feeder was collected at the end of the trial and weighed. Feed waste was not estimated. Feed offered and daily gain averaged 2.94 kg pig^−1^d^−1^ and 0.907 kg pig^−1^d^−1^ respectively.

### 2.4. Soil Sampling

**Figure 1:**
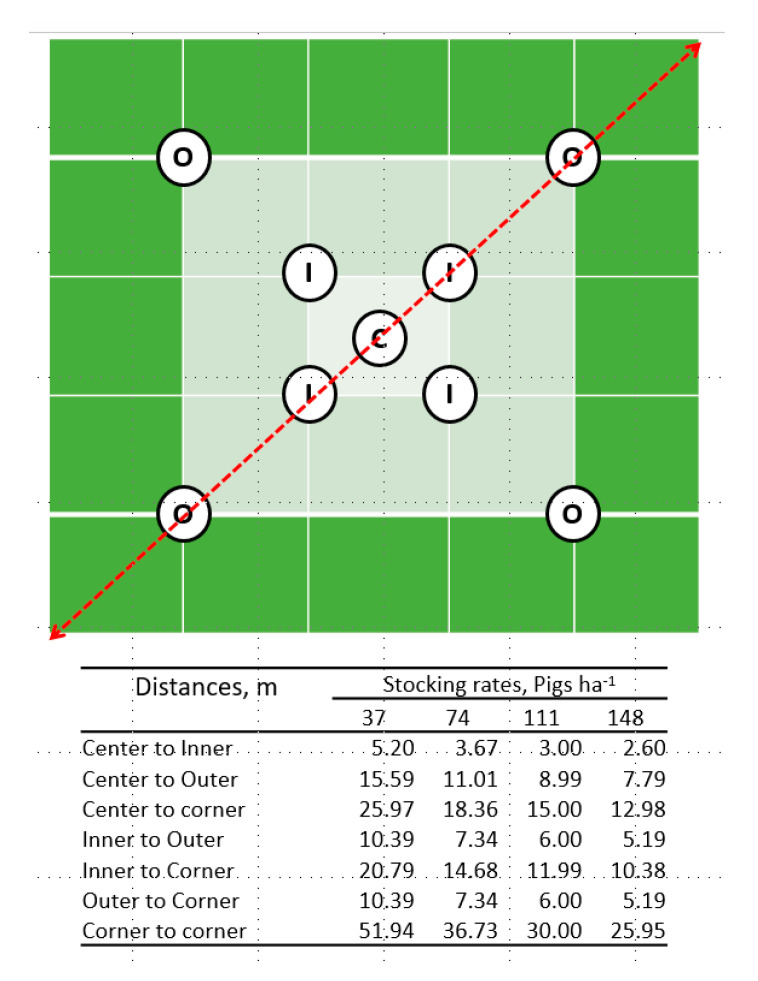
Soil sampling positions. C: Center; I: Inner; O: Outer; Distances among sampling points were estimated using Pythagoras’ theorem [10].

Soil samples were collected on three different dates. The initial sampling (before grazing pigs) followed a grid pattern of 16 equally-spaced sampling points per paddock. Samples were collected to a depth of 90 cm in the increments 0 to 30 and 30 to 90 cm, resulting in two samples per point that were used to establish the baseline soil property condition. The second and third soil samples from the same two soil depth layers were collected after removing the first and second group of pigs from the paddocks, respectively. A different sampling protocol was implemented for these two sampling times: nine sampling points were established within each paddock. Sampling points were classified according to their position relative to the center of the plot to monitor the distribution of soil disturbance within the paddocks: center, inner and outer (1, 4 and 4 samples, respectively, Figure 1). These points were located along transects used to estimate ground cover and positioned proportionally at the same distance and the same location in the different size plots. 

The initial soil samples were collected with a hand-held auger. The soil samples collected after the first group of pigs were collected with a hydraulic sampling device (Model GSRPS Giddings Machine Company, Windsor, CO, USA) that had a 3.2-cm diameter coring tube. The day of sampling the soil was moist and no evidence of soil compaction was observed as the tube penetrated to the 90 cm depth. The coring device allowed for the determination of soil bulk density. Small subsamples of the 45 °C oven-dried samples were collected and dried at 105 °C to determine soil bulk density on a dry basis. The soil was dry when the second group of pigs was removed from the paddocks and the soil was being compacted inside the sampling tube when using the hydraulic sampling device. Therefore, samples were collected with the hand-held auger device used during the initial sampling. Bulk density data corresponding to the second sampling were not included in the analyses. All soil samples collected in the study were oven dried (45 °C) for 24 h, passed through a 2-mm sieve to remove coarse organic fragments and gravel, then ground and sent to the laboratory for nutrient analysis.

### 2.5. Soil Laboratory Analysis

Soil samples were analyzed at the North Carolina Agricultural and Technical State University soil laboratory for bulk density, nitrate (NO_3_-N), ammonium (NH_4_^+^-N), Total-N, PO_4_^−^-P and Total-P concentrations. Soil chemical forms of nitrogen and phosphorous were determined in each soil core sample. The inorganic forms of soil nitrogen (NO_3_^−^-N and NH_4_^+^-N) were extracted with a 2M KCL solution in a 1:10 soil-solution ratio. The concentration of each N form in filtrates were determined spectrophotometrically with a Flow Injection Analyzer (LACHAT Instrument Quick Chem. 8000, HACH, Milwaukee WI, USA). A CHNS/O Analyzer (Perkin-Elmer Model 2400; Perkin Elmer Corporation, Waltham MA, USA) was used to analyze for total nitrogen. Soil phosphorus was extracted with a Bray-1 solution in a 1:7 soil-solution ratio. Concentrations of extractable phosphorus and total phosphorus in the filtrate were determined using inductively coupled plasma optical emission spectroscopy (ICP-OES Perkin-Elmer Optima 3300, Perkin Elmer Corporation, Waltham, MA, USA). The concentration of each nutrient form was converted to the amount in weight on a per hectare volume basis using the soil bulk density determined from the core sample.

### 2.6. Statistical Analysis

The experimental design was a randomized complete block with three field replications. The paddock was considered the experimental unit. Data analyses were performed through analyses of variance and covariance by means of generalized mixed models using the procedure PROC GLIMMIX of SAS/STAT ^®^ Version 9.4 (Copyright 2017© SAS Institute Inc., SAS Campus Drive, Cary, NC, USA) [11]. To study ground cover across time, the model included grazing period, stocking rate, week and their interactions as fixed effects. Block was considered a random effect and week a repeated measure. The model for final ground cover (measured on week 14) incorporated grazing period, stocking rate and their interaction as fixed effects and block as a random effect. Grazing period was analyzed as a repeated measure.

A split-split-split plot analysis was conducted for soil-related variables with grazing periods as the main plot factor, pigs stocking rate (37, 74, 111 and 148 pigs ha^−1^), soil sampling position (center, inner and outer areas within paddock) and soil sampling depth (0 to 30 and 30 to 90 cm) as sub, sub-sub and sub-sub-sub plot factors, respectively. Block was considered a random effect, whereas grazing period, stocking rate, sampling position, soil depth and their interactions were analyzed as fixed effects. Initial soil values were included in the models as covariates. Grazing period and sampling position were analyzed as repeated measures. For all models, the Kenward and Roger option was used to estimate the covariance matrix for the fixed effects and the degrees of freedom for *t*- and F-tests. The compound-symmetry structure, was selected using the Akaike corrected (AICC) and Schwarz’s Bayesian (BIC) criterions. Differences between treatments were determined by the multiple comparison procedure using the SIMULATE adjustment option. Results were considered statistically significant at *p* < 0.05 and are presented as means ± standard error. 

## 3. Results

**Figure 2:**
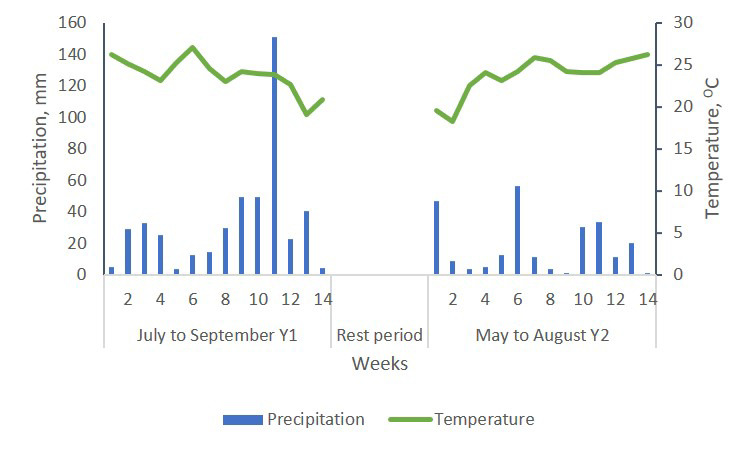
Weekly precipitation and temperature during the experimental period. Source: State climate office of North Carolina, North Carolina State University. CRONOS/ECONet Database [12].

During both grazing periods precipitation (472.4 and 247.4 mm for period 1 and 2, respectively) and temperature (23.8 and 23.8 °C for period 1 and 2, respectively) were favorable for bermudagrass growth (Figure 2). During the 32-week rest-period (time-lapse between the two grazing periods when the paddocks were not grazed), 535.4 mm of rain accumulated and the temperature averaged 10.4 °C. Baseline soil properties from samples collected up to 90 cm depth prior to grazing pigs on the site can be found in Table 2. 

### 3.1. Ground Cover

**Figure 3:**
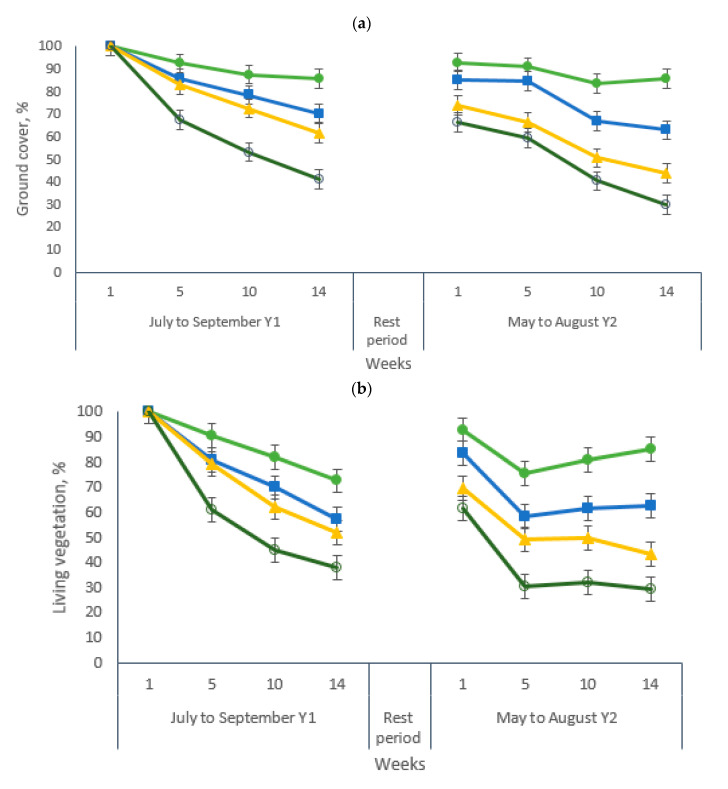

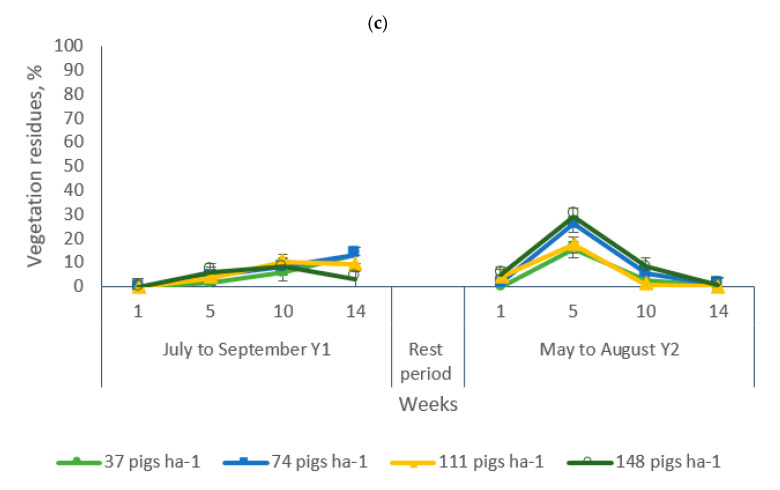
Vegetative ground cover (%) in bermudagrass paddocks grazed with pigs over two 14-week grazing periods. (**a**) Ground cover, (**b**) Living vegetation and (**c**) Vegetation residues. Data are the means of three field replicates. Errors bars represent plus or minus one standard error of the means within the interaction.

Ground cover averaged 80% and 68%, respectively, for grazing periods 1 and 2. Interactions grazing period × stocking rate, grazing period × week and stocking rate × week were observed for ground cover and living vegetation and week and grazing period × week for vegetation residues. The evolution of the ground cover and its components during the two-pig grazing periods are presented in Figure 3a–c. Ground (Figure 3a) and living vegetation cover (Figure 3b) followed a similar decreasing trend from start to end of each grazing period, while vegetation residues cover (Figure 3c) behaved more irregularly, especially during the second grazing period with an initial growth and a final decrease. The ground and living vegetation cover decreased at all stocking rates. However, a greater decrease rate in ground cover was observed for paddocks managed with 148 pigs ha^−1^ compared with those managed with 37 pigs ha^−1^. In the first grazing period, vegetation residues increased from week 1 to week 14 for stocking rates of 37, 74 and 111 pigs ha^−1^ (Figure 3c). In contrast, no cohesive pattern was observed for vegetation residues in paddocks with 148 pigs ha^−1^. A different trend was observed in the second grazing period, where vegetation residues increased greatly during the first 5 weeks, then declined steadily until the end of the period for all stocking rates. Even though the 32-week rest period between grazing periods allowed for the recuperation of the ground cover, no paddock recovered coverage to 100% at the beginning of the second period. 

**Figure 4:**
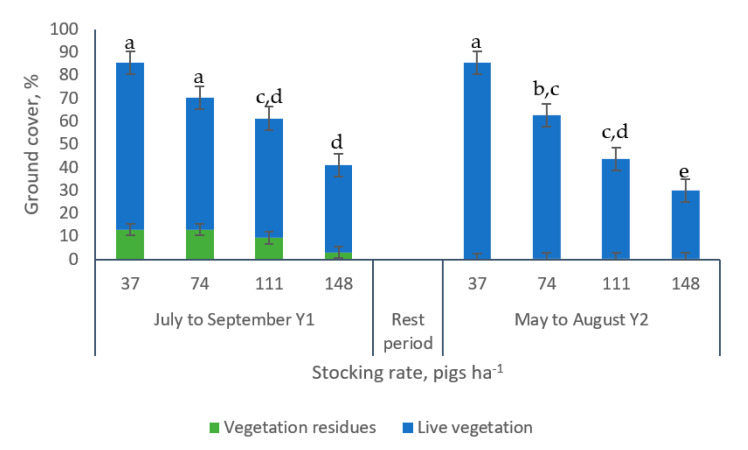
Effect of the interaction grazing period × stocking rate on the ground cover of bermudagrass paddocks at the end of the grazing periods. a–e: means having the same letter are not significantly different at the 5% level of probability as indicated by the Multiple Comparisons test—simulate option. The Letters display does not reflect all significant comparisons. The following additional pairs are significantly different: GP1 SR111 vs. GP2 SR111; GP1 SR148 vs. GP2 SR148; where GP: grazing period and SR: stocking rate. Data are the means of three replicates. Errors bars represent plus or minus one standard error of the means within the interaction.

The analysis corresponding to final ground cover (measured at the end of the grazing periods- week 14), showed significant differences among grazing periods, stocking rates and for the interaction grazing period × stocking rate (Figure 4). In both periods, the greater final ground cover was recorded in paddocks managed with 37 pigs ha^−1^ (110 % and 187 % more ground cover at the end of the grazing periods 1 and 2, respectively) compared to the ground cover found in paddocks managed with 148 pigs ha^−1^. Paddocks managed with a stocking rate of 111 pigs ha^−1^ showed no differences in ground cover with the most intensive treatment (148 pigs ha^−1^) at the end of grazing period 1. However, at the end of grazing period 2, it was possible to detect differences in the ground cover from paddocks managed with 37, 74 and 111 pigs ha^−1^ and the ground cover estimated in paddocks grazed with 148 pigs ha^−1^.

### 3.2. Soil Properties

Initial values for soil properties were similarly distributed across stocking rates but soil bulk density and the concentrations of Total-N, PO_4_^−^-P and Total-P varied along the soil profile, generally showing lesser values deeper in the profile (30 to 90 cm). Previous history of the paddocks, which were intensively grazed with dairy cows, could have influenced the soil values observed in the collected samples. Changes in soil properties over time toward an increase compared to average initial figures were also observed. The inclusion of initial values as covariates in the different models resulted significant for bulk density and PO_4_^−^-P (Table 2).

#### 3.2.1. Bulk Density

**Figure 5:**
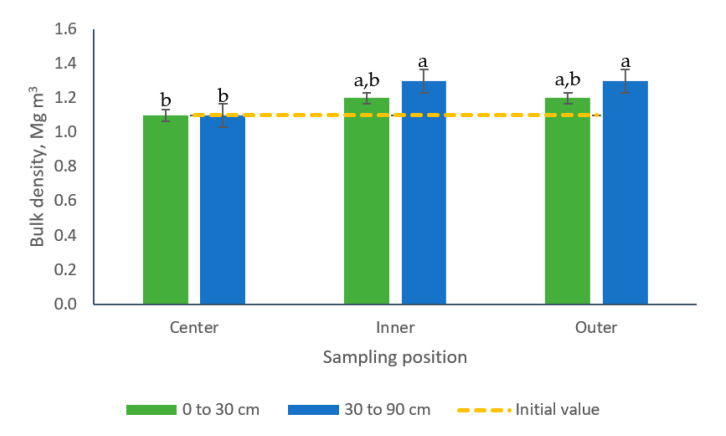
Effect of the interaction sampling position × sampling depth on soil bulk density (Mg m^−3^) of bermudagrass paddocks grazed with pigs during one grazing period. a, b: means having the same letter are not significantly different at the 5% level of probability as indicated by the Multiple Comparisons test—simulate option. Data correspond to the first grazing period and are the means of three field replicates. Errors bars represent plus or minus one standard error of the means within the interaction.

All paddocks had similar bulk density before pig introduction, averaging 1.12 Mg m^−3^ and ranging from 1.0 to 1.25 Mg m^−3^, data not shown. No differences were established in bulk density among samples from paddocks managed with different stocking rates (average 1.20 Mg m^−3^, data not shown). Significant interactions were observed, however, for the interaction sampling position × sampling depth. The samples collected in the center position of the paddocks (both depths) showed lesser bulk density (1.1 Mg m^−3^) compared with those collected in the inner or in the outer positions (30 to 60 cm soil layer) which did not differ and averaged 1.3 Mg m^−3^ (Figure 5).

#### 3.2.2. Soil Nutrients

Results of the analyses conducted to soil nutrients data are presented on Table 2. Greater concentrations (+41%) of Total-N were found in the first grazing period (July to September Y1). Conversely, greater amounts (+87%) of PO_4_^−^-P were recorded in the second grazing period. Stocking rate showed a trend (*p* = 0.0727) toward an increase in NO_3_^−^-N (Table 2), with greater concentrations recorded on paddocks managed with 148 pigs ha^−1^ (+159%) than in paddocks managed with 37 pigs ha^−1^).

Differences in the concentrations of Total-N, PO_4_^−^-P and Total-P were observed among soil samples collected from different positions in the paddocks (Table 2). The inner and outer positions registered 37, 41 and 45% more Total-N, PO_4_^−^-P and Total-P, respectively, compared to samples from the center of the paddocks. No differences were observed, however, among samples collected in the inner and outer positions. Differences related to sampling depth were also observed for these nutrients, with increases of 288% Total-N and 177% Total-P in samples collected from the top soil layer (0 to 30 cm). Conversely, samples from the 30 to 90 cm soil strata contained 154% more PO_4_^−^-P than samples collected in the upper layer (Table 2). 

**Figure 6:**
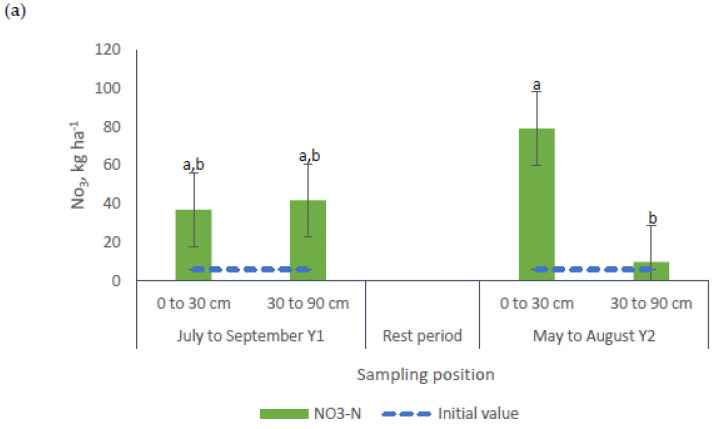

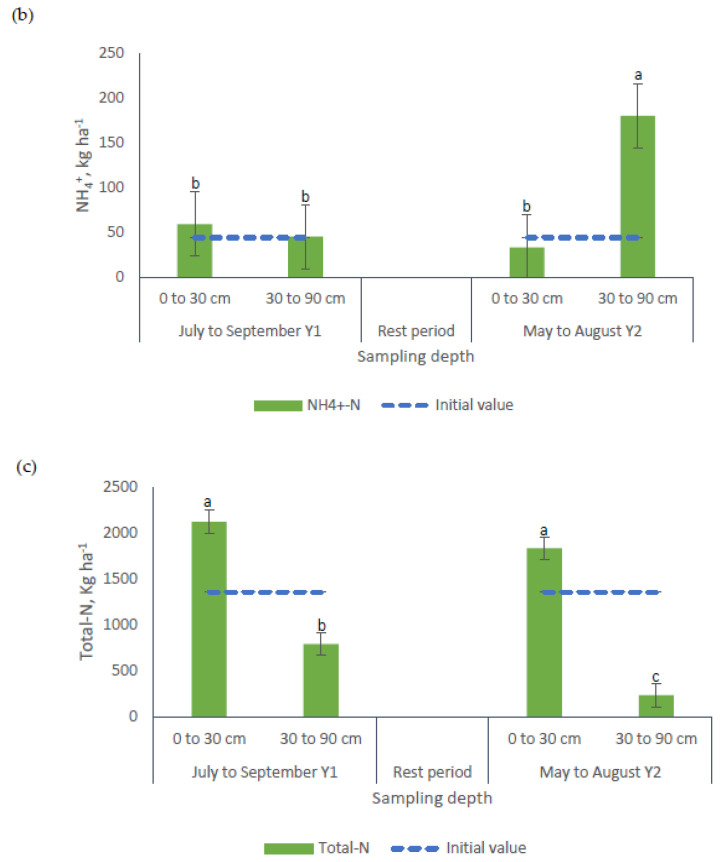
Effects of the interaction grazing period × sampling depth on the concentrations (kg ha^−1^) of (**a**) NO_3_^−^-N, (**b**) NH_4_^+^-N and (**c**) Total-N in soil samples from bermudagrass paddocks managed with different pig stocking rates for two 14-week grazing periods. a, b, c: means having the same letter are not significantly different at the 5% level of probability as indicated by the Multiple Comparisons test—simulate option. Data are the means of three field replicates. Errors bars represent plus or minus one standard error of the means within the interaction.

**Figure 7:**
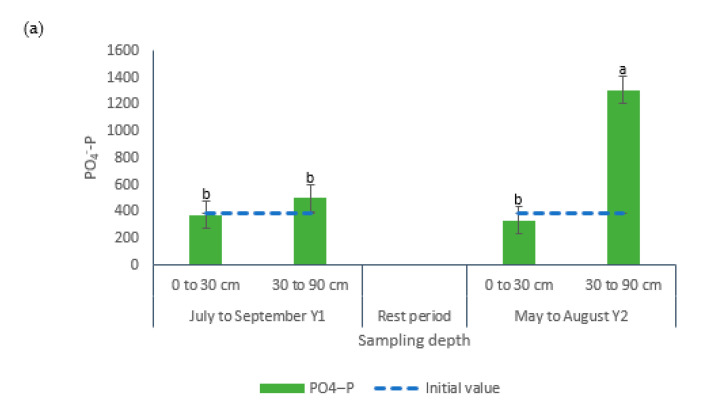

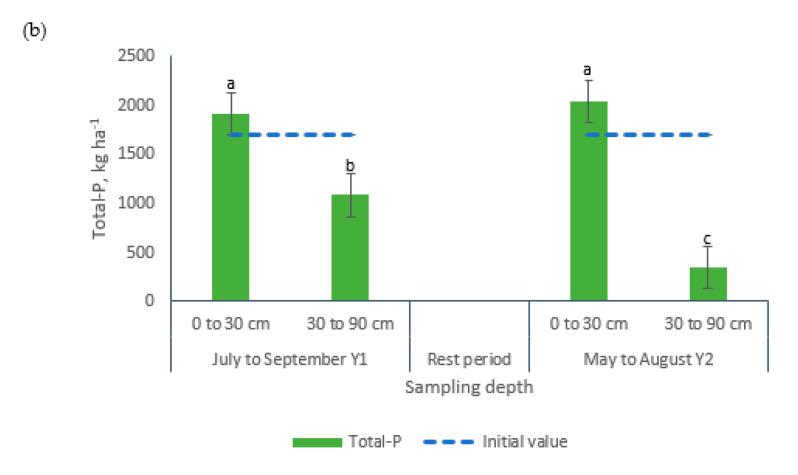
Effects of the interaction grazing period × sampling depth on the concentrations (kg ha^−1^) of (**a**) PO_4_^−^-P and (**b**) Total-P in soil samples from bermudagrass paddocks managed with different pig stocking rates for two 14-week grazing periods. a, b, c: Means having the same letter are not significantly different at the 5% level of probability as indicated by the Multiple Comparisons test—simulate option. Data are the means of three replicates. Errors bars represent plus or minus one standard error of the means within the interaction.

The existence of a trend (*p* = 0.0782) for Total-N was found for the interaction grazing period × sampling position. For all the nutrients under evaluation, differences were observed for the interaction grazing period × sampling depth (Table 2, Figure 6 and Figure 7). No differences were observed in NO_3_^−^-N concentrations in samples collected at different depths during the first grazing period. Conversely, greater concentrations of NO_3_^−^-N (+690%) were found in samples from the 0 to 30 cm layer during the second grazing period (Figure 6a).

During the first grazing period, no differences were observed in the concentration of NH_4_^+^-N among samples from both sampling depths (Figure 6b). During the second grazing period, however, greater NH_4_^+^-N values were found in samples gathered in the bottom layer (30 to 90 cm, a value 440% greater than the value registered in the top layer and 225% greater than the value registered in the first period). Similarly, greater Total-N levels were found in samples from the top soil, which were not different among the grazing periods and averaged 1978 kg Total-N ha^−1^ (Figure 6c). 

Greater concentrations of PO_4_^−^-P were recorded in samples collected following the second grazing period in the 30 to 90 cm soil layer (1308 kg ha^−1^), whereas the other group of samples did not differ and showed 224% less PO_4_^−^-P (Table 2, Figure 7a). The Total-P levels in the upper soil layer did not present differences among grazing periods (average 1976 kg ha^−1^) but that value was greater than the values registered for the samples collected in the bottom soil layer (+82 and 476%) in the first and second grazing period, respectively, (Figure 7b).

**Figure 8:**
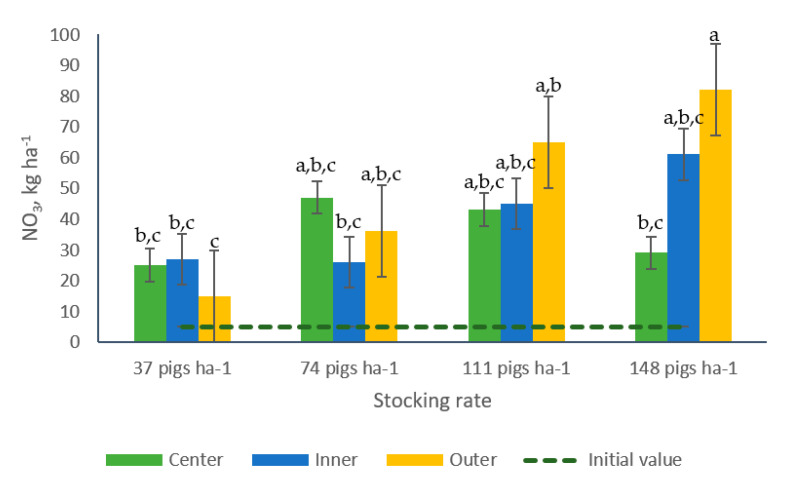
Effect of the interaction stocking rate × sampling position on soil NO_3_^−^-N (kg ha^−1^) in bermudagrass paddocks grazed with pigs during two grazing periods. a, b, c: For each grazing period, means having the same letter are not significantly different at the 5% level of probability as indicated by the Multiple Comparisons test—simulate option. Data are the means of three field replicates. Errors bars represent plus or minus one standard error of the means within the interaction.

A significant interaction stocking rate × sampling position was detected for NO_3_^−^-N concentrations (Table 2). No differences were observed among samples collected from the different sampling positions from paddocks managed with 37, 74 or 111 pigs ha^−1^. Samples from paddocks managed with 148 pigs ha^−1^ collected from the outer position, however, contained greater concentrations of NO_3_^−^-N, 183% more than the concentrations found in samples collected from the center of the same paddocks. Similarly, samples from the outer position from paddocks with 148 pigs ha^−1^ showed greater NO_3_^−^-N (+267%) than the concentrations found in samples collected from the three sampling positions in paddocks managed with 37 pigs ha^−1^ and 215% more that the concentrations found in samples collected from the inner position in paddocks with 74 pigs ha^−1^ (Table 2, Figure 8).

**Figure 9:**
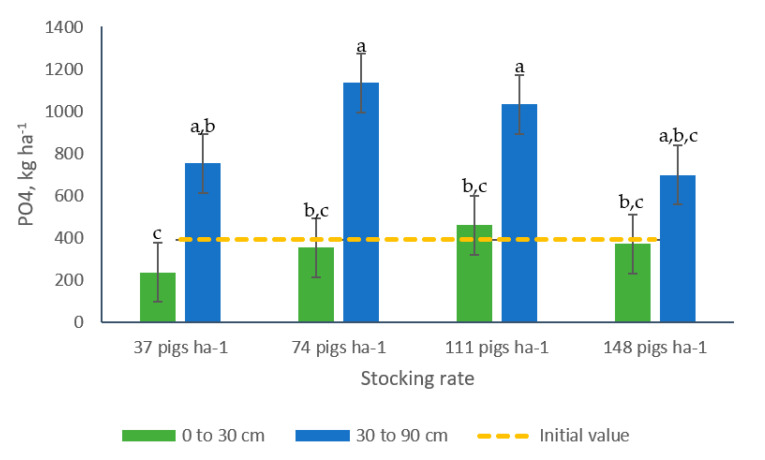
Effect of the interaction stocking rate × sampling depth on soil PO_4_^−^-P (kg ha^−1^) in bermudagrass paddocks grazed with pigs during two grazing periods. a, b, c: Means having the same letter are not significantly different at the 5% level of probability as indicated by the Multiple Comparisons test—simulate option. The Letter display does not reflect all significant comparisons. The following additional pairs are significantly different: SR148 SD30-90 vs. SR148 SD0-30; where SR: stocking rate and SD: sampling depth. Data are the means of three replicates. Errors bars represent plus or minus one standard error of the means within the interaction.

Similarly, the interaction stocking rate × sampling depth proved significant for PO_4_^−^-P (Table 2). Greater concentrations of that nutrient (+154%) were found in the bottom soil layer, with no differences among stocking rate. A comparable pattern was observed in samples collected in the 0 to 30 cm soil profile (Figure 9).

**Figure 10:**
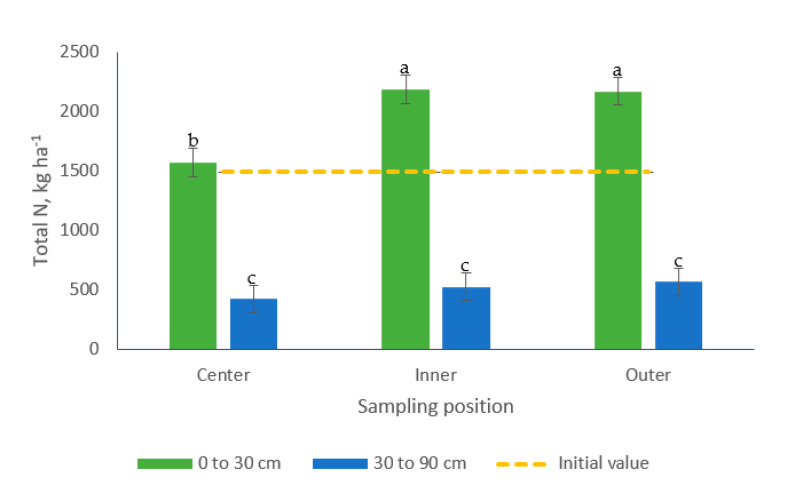
Effect of the interaction sampling position × sampling depth on soil Total-N (kg ha^−1^) in bermudagrass paddocks grazed with pigs during two grazing periods. a, b, c: Means having the same letter are not significantly different at the 5% level of probability as indicated by the Multiple Comparisons test—simulate option. Data are the means of three replicates. Errors bars represent plus or minus one standard error of the means within the interaction.

Total-N patterns resulted in a significant interaction for sampling position × sampling depth (Table 2). Differences in Total-N concentrations were observed in samples from the 0 to 30 cm soil depth. Samples collected from the center of the paddocks contained 39% less Total-N in comparison to samples gathered at the inner and outer positions which were similar (average 2181 kg Total-N ha^−1^). Lesser Total-N concentrations were found in samples collected from the soil bottom layer, with no difference among the positions (average 510 kg Total-N ha^−1^) (Figure 10).

**Figure 11:**
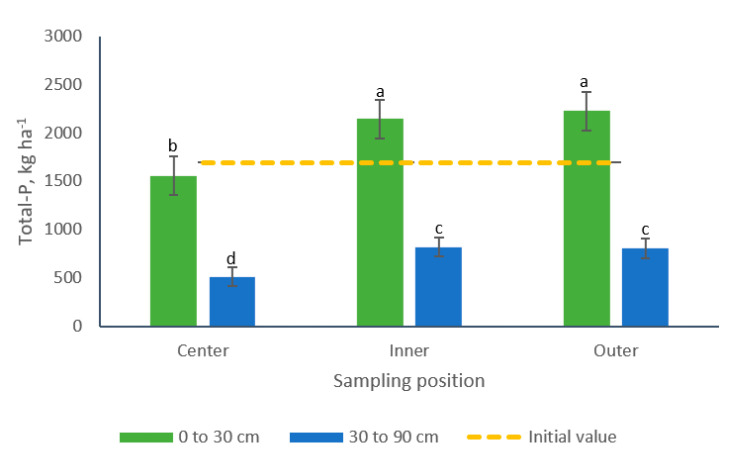
Effect of the interaction sampling position × sampling depth on soil Total-P (kg ha^−1^) in bermudagrass paddocks grazed with pigs during two grazing periods. a, b, c, d: Means having the same letter are not significantly different at the 5% level of probability as indicated by the Multiple Comparisons test—simulate option. Data are the means of three replicates. Errors bars represent plus or minus one standard error of the means within the interaction.

Similarly, a significant interaction for sampling position × sampling depth was detected for Total-P (Table 2). More elevated concentrations of Total-P were found in the top layer of the soil (0 to 30 cm) but similar patterns were observed across positions for both soil depths. Concentrations were similar in the inner and outer positions, averaging 2185 and 813 kg Total-P for the upper and bottom layers, respectively. The latter Total-P concentrations were respectively 40 and 59% greater than those collected from the center of the paddocks (Figure 11).

**Figure 12:**
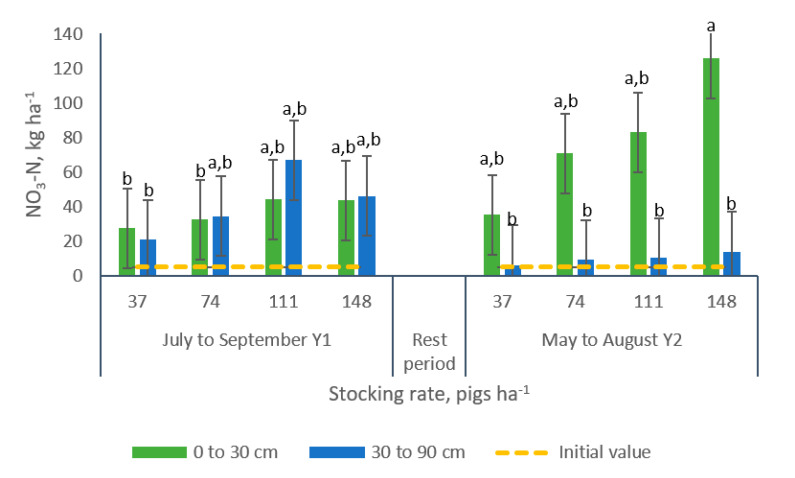
Effect of the interaction grazing period × stocking rate × sampling depth on soil Total-N (kg ha^−1^) in bermudagrass paddocks grazed with pigs during two grazing periods. a, b: means having the same letter are not significantly different at the 5% level of probability as indicated by the Multiple Comparisons test—simulate option. The Letter display does not reflect all significant comparisons. The following additional pairs are significantly different: GP2 SR148 SD0-30 vs. GP2 SR37 SD0-30; where GP: grazing period, SR: stocking rate and SD: sampling depth. Data are the means of three replicates. Errors bars represent plus or minus one standard error of the means within the interaction.

An interaction grazing period × stocking rate × sampling depth was observed for NO_3_^−^-N (Table 2). No differences were noted in samples collected following the first grazing period from paddocks managed with different stocking rate at neither soil layers. However, samples collected after the second grazing period depicted differences among samples collected in the 0 to 30 cm soil layer from paddocks managed with a stocking rate of 148 pigs ha^−1^ compared to samples collected in the 30 to 90 cm soil layer for all the stocking rates under evaluation (Figure 12). 

**Figure 13:**
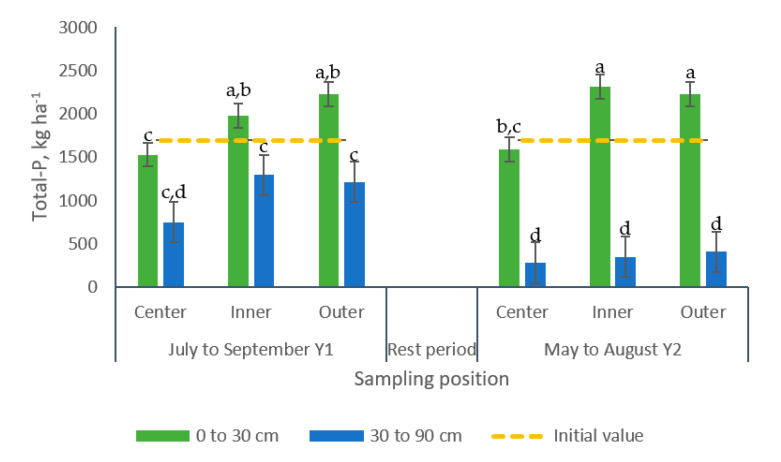
Effect of the interaction grazing period ×sampling position × sampling depth in soil Total-P (kg ha^−1^) in bermudagrass paddocks grazed with pigs during two grazing periods. a, b, c, d: Means having the same letter are not significantly different at the 5% level of probability as indicated by the Multiple Comparisons test—simulate option. The Letter display does not reflect all significant comparisons. The following additional pairs are significantly different: GP1 SP-C SD0-30 vs. GP1 SP-C SD30-90; GP1 SP-I SD30-90 vs. GP1 SP-C SP30-90; GP1 SP-O SD30-90 vs. GP1 SP-C SD30-90; where GP: grazing period; SP: Sampling position, SP-C: center, SP-I: Inner, SP-O: Outer; SD: sampling depth. Data are the means of three replicates. Errors bars represent plus or minus one standard error of the means within the interaction.

An interaction grazing period × sampling position × sampling depth was identified for Total-P levels (Table 2). Both grazing periods showed a similar pattern with greater levels of Total-P in the upper soil layer (0 to 30 cm). In this soil strata, greater concentrations of Total-P were found in samples from the inner and the outer positions of the paddocks, which did not differ and averaged 2101 and 2270 kg ha^−1^ for the first and the second grazing period, respectively). Comparatively, samples collected from the center positions measured 1527 and 1591 kg ha^−1^ for the first and the second grazing period, respectively. Conversely the effect of sampling position was not observed on the bottom soil layer (30 to 90 cm) (Figure 13).

## 4. Discussion

### 4.1. Ground Cover

In pasture pig operations, an appropriate grazing management is necessary to prevent damage to the pasture and, consequently, soil degradation. The amount of ground cover in a certain pasture will be dependent on a variety of factors including the pasture species, climate, soil, pasture management [13] and seasonal variability [14]. The establishment of an adequate stocking rate is a key management component to assure the preservation of ground cover levels required to avoid negative environmental consequences. In 2007, North Carolina United States Department of Agriculture USDA Natural Resource Conservancy Service (NRCS) conservationists established that for conservation purposes, outdoor pig operations should retain a ground cover of 75% or more [15]. Maintaining an appropriate ground cover level offers a wide array of benefits including animal welfare, forage supply, protection against erosion and runoff and ultimately against soil and water pollution [3,7]. Greater nutrient losses (N and P) have been associated with bare soil conditions [16,17]. Similarly, Dourmad and Casabianca [18] highlighted the role of ground cover in minimizing ammonia emissions. The importance of the presence of ground cover in pasture pig systems is associated with its role of trapping and recycling the nutrients deposited through manure [19] and avoiding their losses to the system via runoff and leaching [20].

In the present study, a reduction of ground cover and of its indicators was observed over time. A similar declining response had been reported by Bordeaux et al. [21], using a cover of sudangrass (*Sorghum bicolor*) and a mixture of cereal rye (*Secale cereale*) and ryegrass (*Lollium multiflorum*) at a stocking rate equivalent of 74 pigs ha^−1^. Pigs can have a direct effect on ground cover when they graze but also an indirect effect due to trampling and rooting activities. Trampling has been associated with higher soil compaction, damaged soil aggregates, decreased water infiltration and increased run-off [22,23]. In the study herein, the 32-week rest period between pig grazing periods allowed the ground cover to recover but did not reach the initial ground cover values, a situation that could be an indicator of damage to the vegetation and(or) to the soil structure. Rooting has been linked to a reduction in ground cover as a consequence of the damage to the aerial and radicular portions of the vegetation [24]. It could also be possible that the first grazing period ended too late (last week of September) and the second grazing period started too early (second week of May), therefore not allowing the bermudagrass to completely develop the structures (roots, rhizomes, shoots, tillers, stolons) needed for regrowth and growth, consequently impairing its spring recover [25]. 

The results of this study showed differences in ground cover measured during the last experimental week among paddocks managed under different stocking rates (Figure 4). Only paddocks managed with the lowest stocking rate (37 pigs ha^−1^) maintained the ground cover level recommended by North Carolina NRCS [15]. Similar trends have been observed by Thomsen et al. [4], who reported 31%, 57% and 76% ground cover on mixed pastures of *Poa trivialis*, *Dactylis* spp. and *Bromus* spp. for stocking rates equivalent to 100, 42 and 17 pigs ha^−1^, respectively. Conversely, using growing pigs (20 to 100 kg body weight) managed at a stocking rate equivalent of 100 pigs ha^−1^, Hermansen et al. [26], reported no survival of ground cover. Investigating the effects of stocking rates (244 vs. 86 pigs ha^−1^) and animal categories (growers: 25 to 40 kg body weight vs. finishers: 80 to 105 kg) on *Bromus catharticus* Vahl, Campagna et al. [27], reported significant effects of both factors on the ground cover, with greater ground cover with the lower stocking rate (62% vs. 25% and 65% vs. 45%, respectively). Furthermore, more ground cover damage was observed in paddocks managed with growers at the highest stocking rate. Overstocked conditions will lead to the complete destruction of the vegetation cover with the aforementioned environmental effect.

Greater stocking rates will produce more damage to the cover due to trampling [28]. Additionally, the damage caused to the vegetation is exacerbated when grazing is coupled with rooting. Rooting pressure has been indicated as the cause of loss of vegetation, the starting point for erosion and runoff and a risk factor for ground water pollution [4]. An effect of stocking rate on pigs eating behavior has been reported, with greater frequency of rooting in the low stocking rate treatment (0.57% vs. 0.39% for 100 or 200 pigs ha^−1^, respectively), while more foraging was observed at the high stocking rate (0.17% vs. 0.11% for 200 or 100 pigs ha^−1^, respectively) [29,30]. Accordingly, the strong influence of wild boar rooting in an Argentinian desert ecosystem has been highlighted, resulting in a reduction of the vegetative ground cover and of the species richness [31]. Forage species traits, such as their growth and reproductive habits (erect or prostrate, bunch or sod forming), nutritive value and palatability, can favor rooting behavior [28,32] and affect the ability of the forage to survive [33]. Bermudagrass, with its decumbent growth habit and its rhizomatous and stoloniferous reproductive structures has the potential to withstand the foraging behavior of pigs [33]. Greater deterioration of the vegetation cover as a consequence of rooting and trampling behavior in areas close to the location of the service structures and along paddock fences, have been reported by previous researchers [34,35,36]. In addition to the adjustment of the stocking rate, other practices can be implemented to reduce damage to the vegetative cover. These practices include the selection of forage species that can resist rooting and trampling by pigs [3,5] and avoiding feed restriction [5,28].

### 4.2. Soil Properties

There is evidence regarding the effect of grazing pigs on different soil physical (soil structure [24,35,37], water infiltration [35] bulk density [24,31]) and chemical properties (inorganic N and exchangeable K concentration [38]; N, P, K, Cu, Zn [31,39,40,41]). In addition, the effect of grazing pigs on soil biological properties such as soil respiration, abundance of different functional groups of microorganisms and direct cell counts have been demonstrated [31]. Furthermore, a faster N mineralization rate in wild boar-disturbed patches has also been reported [24,31]. Those patches were linked to physical soil degradation, litter incorporation and increased aeration as a consequence of rooting activity [24,31]. 

The effects of pigs on the soil will vary in function of soil moisture, amount of ground cover, soil texture and topography. The damage caused by pigs on sandy soils is mainly a consequence of rooting behavior which disturbs and mixes the soil superficial strata, whereas in heavy soils the damage is caused by trampling action [28]. More damage will be exerted in sloped conditions [42]. Negative effects on the physical traits of soil such as increases in soil bulk density and compaction, a decline in porosity and reduction in water infiltration will also affect microbiological soil activity by reducing the microbial biomass and changing the microbial community composition [23,28], which in turn influences the decomposition of organic matter and the mineralization of organic N [24]. 

The stocking rate, which is the number of animals grazing, rooting, trampling and defecating per area per a definite time scale [43], is an important factor in the management of pasture systems and may have effects on soil organic matter, soil biological activity, nutrient loads, nutrient cycling, water infiltration, runoff and erosion [37,42,44]. A greater intensity of management and consequently a greater stocking rate, has been associated with a greater effect on ground cover and soil properties [5,36,42]. Greater pig stocking rates involve the use of more supplemental feed, raising the load of nutrients imported to the system and the threat of losses to the environment [6,20,27]. 

Deterioration of physical soil characteristics, such as increases in soil bulk density, have been related to grazing management intensity and animal trampling behavior [22]. Even though an increment (+9.1%) in soil bulk density was reported in this study after one pig grazing period, no difference was associated with stocking rate. These results differed from those reported by Campagna et al. [27] who found lesser soil bulk density in paddocks that were managed with grower vs. finisher pigs at the lesser stocking rate (86 vs. 244 pigs ha^−1^). In the study herein, the soil sand concentration (average 86.9%), the length of the rest period between successive paddock occupation and pig rooting activity could have contributed to counteracting the trampling impact, thus preventing bulk density to reach critical values that could compromise vegetation growth.

The difference in bulk density found in samples from the center of the paddocks compared to those from the inner and outer sampling positions could be a consequence of greater rooting activity in this position where the shelter and water structures were located. The overturning and mixing of soil in this area could have neutralized the trampling effect which was more evident in other areas of the paddocks. Similarly, lesser bulk densities have been recorded in soil patches disturbed by wild boars [31]. These findings and the existence of a significant sampling position × sampling depth interaction, suggest the existence of a spatial heterogeneity in the horizontal and vertical dimensions of the soil attribute measured in paddocks grazed by pigs. 

Other authors have found differences in soil physical characteristics in different sections of paddocks managed with pigs which coincide with the findings of this study. Greater damage to soil structure in the wallow areas followed by the shelter and then the feeding areas has been recorded [37]. Similarly, reductions in medium and narrow pore sizes and increases in wide and fine pore sizes with the impact limited to the 0 to 7 cm soil layer in the proximity of feeders have been reported [35]. Monteverde and Del Pino [45], determined higher soil density in the service areas where the huts were located, than in the grazing areas. Similarly, Bordeaux et al. [21], observed greater compaction on interior subplots of paddocks with permanent shade and drinking structures. Differences among samples collected at different sampling depths, recording greater values with increased depth were also found [45].

Woodland areas with a low density of wild boars supported an increase in soil organic matter concentrations and an improvement in soil properties as a result of the incorporation of manure and plant residues and a stimulation of soil oxygenation and microbiological activity [46]. In the present study, lesser soil nutrient concentrations were associated with the lowest stocking rate. The results obtained herein support previous research that have linked greater stocking rates with increments in soil nitrates and phosphorus and greater N concentrations and N surpluses in soils [27,33,38,45]. Similarly, the adoption of greater stocking rates (40 to 50 pigs ha^−1^ vs. 1 to 2 pigs ha^−1^) in a hilly area of Tuscany resulted in damage of soil physical and chemical properties, including a decrease in carbon concentrations, lesser chemical parameters linked to organic matter and increased mineralization and impaired soil fertility and quality [42]. 

Pig density per unit of area has been identified as one factor that disrupts the excretory behavior of pigs housed indoors [47]. In these systems, a direct relationship between pen size and the size of the area dedicated to defecation has been reported, the latter being larger in larger pens [48]. The size of the paddocks used in the present study (340, 450, 675 vs. 1350 m^2^ for 148, 111 and 74 vs. 37 pigs ha^−1^, respectively) could have limited the expression of the excretory behavior linked with the natural behavior of selecting or avoiding certain areas for excretion [49]. As previously reported, pigs prefer dunging areas 5 to 15 m away from their resting areas [39,50] or in the proximity to fences [51]. In the study herein, there were 5 or more meters between the center and the inner sampling point (Figure 1) only in paddocks with the lowest stocking rate (37 pigs ha^−1^), therefore the urinating or defecating locations could be more a consequence of the paddock design than an expression of the natural behavior of the pigs [52].

The results reported herein validated the influence of the sampling positions on the concentration of soil nutrients and, in consequence, the existence of a spatial pattern in their distribution. Greater concentrations of Total-N, PO_4_^−^-P and Total-P were found in samples from the inner and the outer positions, farther than the shelters and drinking stations but closer to the feeders and fences (Table 2, Figure 10, Figure 11 and Figure 13). The buildup of these nutrients may be related to greater fecal deposition in these areas compared to the center. Defecating pigs tend to walk a longer distance from the resting and feeding areas than when urinating [53], which could lead to differential nutrient accumulation. Irregular distribution of nutrients in paddocks managed with pigs has been previously reported [21,41,54].

In pasture systems, the distribution of wild-boar sow feces followed a spatial aggregated pattern. A binomial distribution has been used to describe the pattern [55]. Similarly, in paddocks of grass and clover, pigs defecation was conducted 50%, 10% and 40% and urination 41%, 27% and 31%, in newly allotted area (50 m^2^ group^−1^ d^−1^), the transfer area and in the service area (including huts, water structure and wallows (200 m^2^ group^−1^), respectively; soil nutrients concentrations followed the same pattern as for defecation [56]. Correspondingly, differences in excretory activity performed during day or night hours have been reported for fattening pigs reared in confinement, who exhibited a preference for defecating in corners away from their feeding and lying areas [57].

It has been estimated that pigs deposit manure in 4% to 24% of the total area of the paddock, which would be the preferred area for defecation, thus concentrating 43% to 95% of the nutrients that would be excreted. Furthermore, Phosphorus concentrations in the topsoil of these preferred areas could be as high as 4 times the concentrations registered in other sections of the paddock [39]. A heterogeneous soil spatial distribution of P has been reported for pig paddocks and has been explained as a consequence of pig excretory behavior, paddock slope and feed input. In addition, more concentration of soil organic-P was found under nearby shade trees [58]. Similar results to those obtained in this study were reported by Bordeaux et al. [21], who indicated that greater concentrations of inorganic N were found in the exterior versus the interior areas of paddocks grazed by growing pigs. 

The interaction grazing period × sampling depth showed significance for all soil nutrients under evaluation (Table 2, Figure 6 and Figure 7). Only NH_4_^+^-N and PO_4_^−^-P presented their greater values in samples collected after the second grazing period and in the bottom soil layer, which may indicate accumulation of these nutrients (Figure 6b and Figure 10a). Perhaps, these nutrients moved from the upper depths to zones below 30 cm, a situation that could represents a water quality environmental risk. Previous research has reported decreases in P concentrations with soil sampling depth [51]. 

The interaction stocking rate and sampling position resulted significant for NO_3_^−^-N. Greater concentrations of this nutrient were found in samples collected in the outer sections (areas contiguous to the fence and where the feeders were located) of paddocks managed with 148 pigs ha^−1^ in comparison with the NO_3_^−^-N concentrations registered in samples collected in paddocks managed with 37 pigs ha^−1^, indistinctly of the position of collection (Table 2, Figure 8). These findings corroborate the results reported by Bordeaux et al. [21], who indicated that greater concentrations of inorganic N were found in the exterior versus the interior areas of paddocks grazed by growing pigs. Similarly, Monteverde and Del Pino [45], reported greater concentrations of NO_3_^−^-N, Available-P and Electrical Conductivity in soil collected from paddocks managed under the greater stocking rate in areas where feeders and drinking points were located. 

Additionally, the results herein point out the existence of an interaction sampling position by sampling depth for bulk density, Total-N and Total-P soil concentrations (Figure 5, Figure 10 and Figure 11). These findings coincide with the results of Scharifi et al. [41] who suggested that the concentration of soil nutrients in paddocks individually grazed with lactating sows showed a marked spatial (horizontal and vertical) pattern along four areas. According to these authors, the pattern varied among soil nutrients and also among the form of the nutrients, which presented an uneven pattern in both the vertical and horizontal dimensions of the soils. For example, the concentration for P (Olsen) in the 0 to 15 cm soil layer was higher in the feeding area followed by the wallow, then the huts and finally the grazing areas, while in the 15 to 30 cm soil layer the pattern was huts followed by wallows, then feeding areas and last grazing areas.

Pigs tend to concentrate their activities in certain areas of the paddocks such as the vicinity of shelters, shades, feeders, water structures and fences. As a consequence of grazing, rooting and trampling behavior, the survival of the vegetation in these areas may be compromised. Damaged ground cover can result in a reduction of nutrient removal and in an increased potential of runoff and erosion. Higher numbers of animals can increase the eventuality of environmental hazards. To minimize the effects that grazing pigs could have on the environment, best management practices of animals and pastures should be implemented. Adjustment of stocking rates [20,28,35,54,59], service infrastructure rotation [20,21,35] and adoption of rotational stocking [54,59] are measures that have proven effective. 

## 5. Conclusions

This study provides insight into the effects of the intensity of use of bermudagrass paddocks by growing-finishing pigs. Greater ground cover and lesser soil nutrient concentrations were found in the paddocks managed with the lesser pig-stocking rates. Additionally, soil properties differed among sampling points and depths, with differences in the spatial pattern followed for each soil traits. The results suggest that a moderate stocking rate would likely be an effective management tool to improve the environmental sustainability of pasture-based pig production systems. Considering conservation goals and the results obtained, stocking rates for growing to finishing pigs should not be higher than 37 pigs ha^−1^. Further research is needed to examine the long-term effects of pig stocking rates on the productivity and sustainability of pasture-based pig production systems.

## Figures and Tables

**Table 1:** Stocking rates under evaluation.

Stocking Rate		Paddock Size
Pigs ha^−1^	Kg BW ha^−1^	m^2^
37	2689	1350
74	5378	675
111	8067	450
148	10756	340

Kg BW: kg body weight estimated as a function of the average weight. (Final weight—Initial weight) of the two groups of pigs.

**Table 2:** Soil nutrients (kg ha^−1^) in bermudagrass paddocks grazed with pigs for two 14-week grazing periods.

		NO_3_^−^-N	NH_4_^+^-N	Total-N	PO_4_^−^-P	Total-P
		(kg ha^−1^)				
**INITIAL VALUE**		5 ± 1	41 ± 2	1493 ± 49	391 ± 21	1695 ± 91
**MEAN**		42 ± 4	79 ± 9	1244 ± 76	630 ± 44	1345 ± 73
**Grazing Period GP**	**July to Sept Y1**	39	52	1456 a	438 b	1496
	**May to Aug Y2**	44	107	1032 b	821 a	1193
	**SE**	17	31	101	86	193
	**Prob**	0.8338	0.2068	0.0398	0.0263	0.2724
**Stocking Rate SR**	**37 pigs ha^−1^**	22 b	55	1126.0	495	1260
	**74 pigs ha^−1^**	37 a,b	83	1313.0	744	1395
	**111 pigs ha^−1^**	51 a,b	85	1232.0	746	1355
	**148 pigs ha^−1^**	57 a	94	1304.0	535	1369
	**SE**	15	31	140	126	203
	***P***	0.0727	0.376	0.7537	0.3708	0.9075
**Sampling Position SP**	**CENTER**	36	97	1000 b	495 b	1035 b
	**INNER**	40	78	1359 a	710 a	1482 a
	**OUTER**	49	63	1372 a	685 a	1517 a
	**SE**	14	29	87	79	170
	***P***	0.3796	0.1213	<0.0001	<0.0001	<0.0001
**Sampling Depth SD**	**0–30 cm**	58	46	1978 a	356 b	1976 a
	**30–90 cm**	26	113	510 b	904 a	713 b
	**SE**	16	33	99	90	189
	***P***	0.1735	0.1839	<0.0001	0.0062	0.0213
**GP × SR**	***P***	0.5200	0.2101	0.9816	0.8352	0.6534
**GP × SP**	***P***	0.6109	0.3297	0.0782	0.2183	0.4372
**GP × SD**	***P***	<0.0001	<0.0001	0.0365	<0.0001	<0.0001
**SR × SP**	***P***	0.0236	0.5374	0.4181	0.5919	0.7388
**SR × SD**	***P***	0.2578	0.3246	0.2481	0.0028	0.7049
**SP × SD**	***P***	0.8504	0.2825	0.0022	0.2670	0.0501
**GP × SR × SP**	***P***	0.3333	0.5550	0.5790	0.2552	0.8892
**GP × SR × SD**	***P***	0.0416	0.2979	0.9975	0.6200	0.1073
**GP × SP × SD**	***P***	0.5591	0.4674	0.9936	0.0891	0.0554
**SR × SP × SD**	***P***	0.4990	0.5918	0.9789	0.1221	0.9884
**GP × SR × SP × SD**	***P***	0.9031	0.4125	0.5531	0.2570	0.9257
**Initial value—covariate**	***P***	0.3991	0.8028	0.8942	0.0002	0.2370

NO_3_^−^-N: Nitrate-N, NH_4_^+^-N: Ammonium-N, Total-N, PO_4_^−^-P: Phosphate-P, Total-P, SE: Standard Error, *P*: Probability, a, b: means having the same letter in common are not significantly different at the 5% level of probability as indicated by Multiple Comparisons test using the Simulate option. Data are the means of three field replicates.
